# Efficacy and Safety of Vildagliptin Sustained-Release and Metformin Sustained-Release Fixed-Dose Combination in Indian Patients With Type 2 Diabetes Mellitus: A Real-World Perspective

**DOI:** 10.7759/cureus.82549

**Published:** 2025-04-18

**Authors:** Archana Sarda, Bhavana Sosale, Balaji Jagan Mohan, Snehal Tanna, Vipul Gupta, Sanjay Gupta, Deepak Langade, Rahul Kotwal, Vivek Kolapkar, Kamlesh Patel

**Affiliations:** 1 Department of Diabetology, Sarda Centre For Diabetes and Selfcare, Chhatrapati Sambhaji Nagar, IND; 2 Department of Diabetology, Diacon Hospital, Retina Institute of Karnataka, Bengaluru, IND; 3 Department of Diabetology, Apollo Sugar Clinic, Bengaluru, IND; 4 Department of Diabetology, Jupiter Hospital, Thane, IND; 5 Department of Diabetology, Gupta Ultrasound and Heart Care Centre, New Delhi, IND; 6 Department of Diabetology, S.G. Diabetes Centre, Delhi, IND; 7 Pharmacology, D. Y. Patil University School of Medicine, Navi Mumbai, IND; 8 Department of Medical Affairs, Lupin Limited, Mumbai, IND

**Keywords:** early combination, fixed-dose combination, glycemic control, treatment-naïve, vildagliptin sr

## Abstract

Introduction

The efficacy of vildagliptin as an add-on to metformin has been demonstrated in type 2 diabetes mellitus (T2DM) patients inadequately controlled on metformin monotherapy, as well as in early combination therapy for newly diagnosed T2DM patients. However, there is limited evidence on the efficacy and safety of a fixed-dose combination (FDC) of vildagliptin sustained-release (SR) and metformin SR in these patient groups. This study evaluated the efficacy and safety of an FDC of vildagliptin SR and metformin SR in patients with T2DM in real-world clinical settings across India.

Methods

A retrospective, multicenter, post-marketing study was conducted. Data from T2DM patients across 95 centers in India were collected and analyzed retrospectively. The study included adults (18-65 years) diagnosed with T2DM who were either newly diagnosed or inadequately controlled on existing therapy and were prescribed vildagliptin SR 100 mg and metformin SR 500/1000 mg FDC at the discretion of their treating physician. Primary outcomes assessed were reductions in HbA1c (%), fasting plasma glucose (FPG), and postprandial plasma glucose (PPG) from baseline to day 90.

Results

Data from 1,090 patients were included for analysis. The mean age of the patients was 54.46 years, and the mean baseline HbA1c was 8.19 ± 1.22%. The FDC of vildagliptin 100 mg SR and metformin 500 mg SR was prescribed to 89.26% of patients, while the FDC of vildagliptin 100 mg SR and metformin 1000 mg SR was prescribed to 10.73%. The overall study population showed a reduction of −0.81 ± 1.00% in HbA1c (p < 0.0001). Significant reductions in FPG of −28.61 ± 35.15 mg/dL (p < 0.0001) and in PPG of −42.32 ± 52.19 mg/dL (p < 0.0001) were observed. No serious adverse events were reported during the study.

Conclusion

The FDC of vildagliptin SR and metformin SR was effective, safe, and well-tolerated for managing T2DM in patients who were either newly diagnosed or inadequately controlled on other antidiabetic agents in Indian real-world settings.

## Introduction

Diabetes represents a significant universal health challenge. According to the IDF (International Diabetes Federation) Diabetes Atlas, the global prevalence of diabetes has reached pandemic proportions. Recent estimates from the 10th edition indicate that approximately 537 million adults worldwide are affected, accounting for 10.5% of individuals aged between 20 and 79 years. Projections suggest a continued upward trajectory, expected to reach 643 million by 2030 and 783 million by 2045. IDF estimates also suggest a rising trend in diabetes incidence among Southeast Asian nations. In 2021, the incidence of diabetes in the world, Southeast Asia, and India was 10.5%, 8.8%, and 9.6%, respectively [1,2]. India has witnessed a pronounced surge in diabetes and pre-diabetes cases in recent years. Pooled data from numerous studies reveal a substantial burden of diabetes across rural and urban regions, gradually narrowing the differences between urban and rural populations [3]. A National Non-Communicable Disease Monitoring Survey highlighted this trend, reporting a prevalence of 9.3% for diabetes and 24.5% for impaired fasting blood glucose in India [4]. According to a recent Indian Council of Medical Research-India Diabetes (ICMR-INDIAB-17) study published in 2023, the overall prevalence of diabetes in India is 11.8%, with the prevalence of pre-diabetes being 15.3% [5].

Dipeptidyl peptidase-4 (DPP-4) inhibitors are often used as a second-line therapy added to metformin. They have increasingly replaced sulfonylureas as the second-line therapy and are available in fixed-dose combinations with metformin [6]. Despite the evolving role of sodium-glucose transport protein 2 (SGLT2) inhibitors as preferred second-line drugs, with glucagon-like peptide-1 receptor agonists (GLP-1 RAs) as an alternative in particular sets of patient populations, the role of DPP-4 inhibitors remains significant due to their favorable efficacy and tolerability profile [7]. DPP-4 inhibitors have a favorable safety profile, with a low risk of hypoglycemia or weight gain, and are safe in terms of cardiorenal outcomes. Additionally, they are a suitable choice for elderly individuals with type 2 diabetes mellitus (T2DM) [7] and can be used in T2DM patients regardless of comorbidities such as atherosclerotic cardiovascular disease (ASCVD), heart failure (HF), or chronic kidney disease (CKD) [8]. Vildagliptin is a potent, selective, and reversible DPP-4 inhibitor, providing a longer duration of action. Supported by randomized controlled trials, meta-analyses, and over 15 years of clinical use, vildagliptin has been studied extensively in T2DM management as monotherapy, dual therapy, and triple therapy, as well as in combination with insulin. It has also been evaluated in patients with multiple comorbidities.

Compared to other DPP-4 inhibitors, vildagliptin has a more favorable effect on reducing glycemic variability [9]. Vildagliptin is a commonly prescribed DPP-4 inhibitor in T2DM patients owing to its effectiveness in reducing glycated hemoglobin (HbA1c) levels, reduced mean amplitude of glycemic excursion (MAGE), low risk of hypoglycemia, and weight neutrality [10]. The efficacy of the vildagliptin and metformin fixed-dose combination has been demonstrated in T2DM patients inadequately controlled by metformin monotherapy, as well as in early combination therapy in treatment-naïve T2DM patients. Vildagliptin, as an add-on therapy to metformin, improves glycemic control with a significant decrease in fasting plasma glucose (FPG), postprandial glucose (PPG), and HbA1c levels, with a low risk of weight gain and hypoglycemia [11,12].

The management of diabetes is complex and necessitates a compliance-oriented approach to simplify treatment regimens. One effective strategy is the use of once-daily (QD) combination pills to reduce the complexity of the drug regimen and enhance adherence to antidiabetic therapy [13]. Vildagliptin sustained-release (SR) and metformin SR formulations offer promising solutions in this regard. Vildagliptin, when administered as a QD 100 mg SR formulation, has been shown to provide glycemic control comparable to the traditional 50 mg twice-daily (BD) regimen. Studies have established the bioequivalence of vildagliptin 100 mg SR QD with the 50 mg BD regimen, achieving similar DPP-4 inhibition (80%) over 24 hours and thereby improving glycemic control while reducing dosing frequency [13]. Similarly, metformin SR addresses the limitations of immediate-release (IR) metformin by delaying the time to peak plasma concentrations, increasing gastric residence time, and ensuring slower absorption from the upper gastrointestinal tract. This results in improved gastrointestinal tolerability and the convenience of QD dosing [14]. Combining these SR formulations of vildagliptin and metformin could, therefore, significantly reduce the pill burden and improve patient adherence in diabetes management [13,14].

This study aimed to evaluate the efficacy and safety of an FDC of vildagliptin SR 100 mg and metformin SR 500/1000 mg in real-world clinical settings across India. The study specifically focused on two patient populations: newly diagnosed T2DM patients and those inadequately controlled on metformin monotherapy. The assessment included clinical outcomes such as reductions in HbA1c, FPG, and PPG, along with the safety profile of the FDC. Given the need for effective and convenient treatment strategies for glycemic control, this study provides real-world evidence on the use of this FDC in routine clinical practice.

## Materials and methods

Study design and ethical considerations

This was a retrospective, multicenter, real-world, post-marketing study. Patient data were collected retrospectively from 95 centers across India. The study was approved by the Institutional Ethics Committee (IEC) of D. Y. Patil University School of Medicine, Navi Mumbai (Ref. no. DYP/IECBH/2023/123 dated May 13, 2023). The study was conducted in accordance with the Indian Council of Medical Research (ICMR) and International Council on Harmonization (ICH) guidelines for Good Clinical Practice, as well as the principles of the Declaration of Helsinki. Written informed consent was obtained from all participants before inclusion.

Patient population

The study included adult T2DM patients aged 18 to 65 years, either newly diagnosed, treatment-naive, or uncontrolled on existing therapy, who were prescribed vildagliptin SR 100 mg and metformin SR 500/1000 mg FDC with 90-day follow-up data available. Exclusion criteria comprised individuals with type 1 diabetes mellitus, gestational diabetes, pancreatic diabetes, post-transplantation diabetes, malignancies, metabolic diseases, moderate-to-severe renal or hepatic impairment, and known hypersensitivity to study drugs. Patients with incomplete data or medical records were excluded from the study.

Study outcome measures

The primary efficacy outcomes of this study were assessed by evaluating the reduction in glycemic parameters, including HbA1c, FPG, and PPG, from baseline to day 90 of the study period. Secondary efficacy outcomes aimed to provide subgroup analysis of HbA1c reduction in correlation with various patient characteristics. These included stratification based on age groups, body mass index (BMI) categories, duration of T2DM, and the presence of comorbidities such as hypertension, dyslipidemia, and cardiovascular and renal disease. Safety and tolerability outcomes included the reported incidence of any adverse events. Adverse events, including hypoglycemia, were recorded based on patient self-reporting and clinical documentation. Hypoglycemia was defined as a blood glucose level <70 mg/dL. Other adverse events were assessed during follow-up visits.

Data collection and standardization

Data were collected retrospectively from patient medical records maintained at participating centers. To ensure consistency across sites, standardized case report forms (CRFs) were used. Investigators followed predefined protocols to extract data, including baseline demographics, glycemic parameters, treatment history, and adverse events. Site-to-site variations in data collection methods were minimized through periodic quality checks.

Statistical analysis

Descriptive statistics were used to summarize baseline characteristics, with continuous variables presented as mean and standard deviation, and categorical variables expressed as frequencies and percentages. Data from all participating centers were pooled, and analysis was performed using the paired t-test for within-group comparisons and ANOVA for between-group comparisons. Missing values for key parameters were handled using appropriate imputation techniques. For patients with missing glycemic data at follow-up, last observation carried forward (LOCF) was applied, while missing safety data were analyzed descriptively. Sensitivity analyses were performed to assess the impact of missing data on study outcomes. A p-value <0.05 was considered statistically significant.

## Results

Patient demographics

The data of a total of 3956 T2DM patients who were prescribed vildagliptin SR and metformin SR FDC were screened, of which 1,090 T2DM patients having at least 90-day follow-up visit data were identified and were considered for analysis. Table 1 presents the baseline characteristics of the patients included in this analysis.

**Table 1:** Baseline and demographic characteristics SD: standard deviation, ASCVD: atherosclerotic cardiovascular disease.

Parameter	Total (n=1090)
Age in years, mean ± SD	54.46 ± 12.26
Participant ≤ 60 years, mean ± SD (n=754)	48.36 ± 8.63
Participant > 60 years, mean ± SD (n=336)	68.15 ± 7.00
Male % (n)	62.4% (681)
Female % (n)	37.5% (409)
Weight in kg, mean ± SD	72.88 ± 12.23
BMI in kg/m^2^, mean ± SD	27.82 ± 4.69
Smokers % (n)	24.4% (266)
Alcohol % (n)	28.99% (316)
Duration of diabetes in years, mean ± SD	4.85 ± 5.85)
<1 year, % (n)	14.13% (154)
1 to <5 years, % (n)	48.07% (524)
5 to <10 years, % (n)	25.69% (280)
≥10 years, % (n)	12.11% (132)
Baseline glycemic parameters	
HbA1c %, mean ± SD	8.19 ± 1.22
Fasting plasma glucose mg/dL, mean ± SD	171.72 ± 44.16
Postprandial plasma glucose mg/dL, mean ± SD	240.17 ± 63.61
Comorbid conditions	
Absent, % (n)	86.7% (945)
Present, % (n)	13.3% (145)
Hypertension, % (n)	12.75% (139)
Dyslipidemia, % (n)	5.5% (60)
ASCVD, % (n)	0.64% (7)
Heart failure, % (n)	0.46% (5)
Mild renal disease, % (n)	0.18% (2)
Active thyroid disease, % (n)	1.01% (11)

The majority of patients (62.2%) had a duration of T2DM of less than five years. Hypertension was the most commonly reported comorbidity, followed by dyslipidemia (Table 1).

Data for medication history were available for 21.1% of patients, of whom the majority were prescribed metformin (16.96%) and sulfonylurea (10%). The analysis of co-medications revealed that, in addition to antidiabetic agents, patients were also co-prescribed antihypertensive medications, lipid-lowering therapies, and antiplatelet agents. Other common co-medications included antidepressants such as benzodiazepines, pregabalin for neuropathic pain, thyroxine for hypothyroidism, proton pump inhibitors, as well as multivitamins and mineral supplements.

Efficacy outcomes

In the overall patient cohort, HbA1c (%) was reduced by 0.81 ± 1.00 at day 90 compared to baseline (p<0.0001). Both FPG and PPG levels also decreased significantly at day 90 compared to baseline (p<0.0001) (Table 2).

**Table 2:** Reduction in glycemic parameters from baseline to day 90 (n = 1090) Data presented as mean ± SD. *p<0.0001 baseline vs day 90 (paired test). FPG: fasting plasma glucose; PPG: postprandial glucose test; HbA1C: glycated hemoglobin.

Parameters	Mean ± SD	t	p*
FPG (mg/dL)			
Baseline	171.72 ± 44.16	26.873	<0.0001
Day 90	143.11 ± 40.80		
Change from baseline	-28.61 ± 35.15		
PPG (mg/dL)			
Baseline	240.17 ± 63.61	26.772	<0.0001
Day 90	197.84 ± 57.44		
Change from baseline	-42.32 ± 52.19		
HbA1C (%)			
Baseline	8.19 ± 1.22	26.766	<0.0001
Day 90	7.39 ± 0.89		
Change from baseline	-0.81 ± 1.00		

Subgroup analysis based on age showed that HbA1c reduction was higher in patients aged ≤60 years compared to those aged >60 years. Patients exhibited a progressive increase in HbA1c reduction across BMI categories, with the highest reduction observed in obese (≥25 kg/m²) patients, followed by overweight (23 to 24.9 kg/m²), normal weight (18.5 to 22.9 kg/m²), and underweight (<18.5 kg/m²) individuals. Furthermore, there was a significant reduction in HbA1c (p < 0.0001) based on the duration of T2DM and the presence or absence of comorbidities (Table 3).

**Table 3:** Reduction in glycemic parameters in subgroup categories from baseline to day 90 Data presented as mean ± SD. *p<0.0001 between group comparisons (ANOVA). ^#^p<0.0001 within group-paired t-test. Underweight: <18.5 kg/m^2^; Normal: 18.5 to 22.9 kg/m^2^; Overweight: 23 to 24.9 kg/m^2^; Obese: >25 kg/m^2^. BMI: body mass index; HbA1C: glycated hemoglobin.

Patient characteristics	N	HbA1c at Visit 1 (Baseline)			HbA1c at Visit 2 (Day 90)			Change in HbA1c from baseline				
		Mean ± SD	F	p*	Mean ± SD	F	p*	Mean ± SD	F	p*	t	p^#^
Age (years)												
≤60 years	754	8.23 ± 1.21			7.39 ± 0.89			-0.84 ± 1.00			23.142	<0.0001
>60 years	336	8.11 ± 1.24			7.38 ± 0.88			-0.73 ± 0.99			13.571	<0.0001
Total	1090	8.19 ± 1.22	2.096	0.148	7.39 ± 0.89	0.021	0.886	-0.81 ± 1.00	0.100	0.100	26.766	<0.0001
BMI (kg/m^2^)												
Underweight	14	8.41 ± 1.86			7.91 ± 1.57			-0.51 ± 1.55			1.223	0.243
Normal weight	128	7.88 ± 1.32			7.17 ± 0.81			-0.70 ± 1.17			6.820	<0.0001
Overweight	171	8.04 ± 1.18			7.30 ± 0.96			-0.74 ± 0.97			9.939	<0.0001
Obesity	777	8.28 ± 1.19			7.43 ± 0.86			-0.85 ± 0.96			24.612	<0.0001
Total	1090	8.19 ± 1.22	5.280	0.001	7.39 ± 0.89	5.402	0.001	-0.81 ± 1.00	1.560	0.197	26.766	<0.0001
Duration of T2DM												
<1 years	154	8.34 ± 1.35			7.16 ± 0.85			-1.18 ± 1.07			13.692	<0.0001
1 to <5 years	524	8.13 ± 1.09			7.42 ± 0.82			-0.71 ± 0.87			18.567	<0.0001
5 to <10 years	280	8.15 ± 1.33			7.41 ± 0.93			-0.74 ± 1.12			11.065	<0.0001
≥10 years	132	8.37 ± 1.31			7.46 ± 1.05			-0.91 ± 0.99			10.576	<0.0001
Total	1090	8.19 ± 1.22	2.362	0.070	7.39 ± 0.89	4.059	0.007	-0.81 ± 1.00	10.263	<0.0001	26.766	<0.0001
Comorbidity												
Present	145	8.70 ± 1.46			7.39 ± 1.03			-1.31 ± 1.19			13.199	<0.0001
Absent	945	8.12 ± 1.16			7.39 ± 0.86			-0.73 ± 0.94			23.907	<0.0001
Total	1090	8.19 ± 1.22	29.567	<0.0001	7.39 ± 0.89	0.009	0.926	-0.81 ± 1.00	43.936	<0.0001	26.766	<0.0001

Safety outcomes

Side effects were reported in three patients, of which dizziness was reported in one patient, hypoglycemia in one patient, and itching in one patient. No serious adverse events were reported during the study period.

## Discussion

Insulin resistance and dysfunction of pancreatic islets are key pathogenetic pathways of T2DM. Metformin is known to attenuate insulin resistance, while DPP-4 inhibitors improve islet dysfunction by preserving the biological activity of endogenous GLP-1. DPP-4 inhibitors also suppress glucagon secretion from pancreatic α-cells. Additionally, recent research suggests that metformin enhances the physiological effect of GLP-1 by increasing its secretion, and DPP-4 inhibitors also positively impact insulin sensitivity in patients with T2DM.

Due to the multiple pathophysiological abnormalities and the progressive nature of T2DM, therapy intensification with drug combinations is typically required over time. Recent guidelines suggest earlier use of pharmacologic combinations to meet stringent glycemic targets while avoiding adverse events, particularly hypoglycemia [15]. The American Diabetes Association (ADA) 2024 guidelines recommend considering early combination therapy over a stepwise approach in patients presenting with HbA1c levels 1.5-2% above the target, to shorten the time to attainment of individualized treatment goals [8].

In line with current evidence, the South Asian Health Foundation consensus also suggests DPP-4 inhibitors as the preferred class of medication for South Asian individuals with T2DM over sulfonylureas, due to the lower BMI cut-off for obesity and their favorable hypoglycemia risk profile during fasting. The consensus advocates for early and proactive management approaches in this patient population, aiming to reduce the risk of microvascular and macrovascular complications [16].

Clinical inertia often delays the intensification of treatment after metformin failure, resulting in suboptimal glycemic control and causing many patients to experience multiple treatment failures. Also, metformin alone is often insufficient for patients with HbA1c levels above 8.0-8.5% at diagnosis. Early combination therapy addresses this problem by targeting multiple pathophysiological abnormalities of the disease with complementary drug actions. Implementing early combination therapy can also modify the disease's progression, offering more durable glycemic control and potentially altering the clinical course of T2DM [17]. Hence, combining DPP-4 inhibitors with metformin produces an additive or potentially synergistic effect on metabolic regulation in individuals with T2DM, making it a rational combination [18].

Multiple clinical trials have shown that combination therapy with DPP-4 inhibitors and metformin, either as an add-on therapy or as an initial combination, is efficacious and safe in patients with T2DM [18]. Studies have demonstrated the clinical effectiveness of vildagliptin administered in combination with metformin as first-line or second-line treatment in newly diagnosed T2DM patients and in those inadequately controlled on metformin monotherapy [19].

The INITIAL trial evaluated vildagliptin plus metformin in drug-naive T2DM patients, including those with comorbidities such as dyslipidemia, hypertension, and obesity. The trial showed that in a relatively young, drug-naive Asian population with T2DM, high baseline HbA1c levels, and cardiovascular risk factors, vildagliptin/metformin combination therapy significantly reduced HbA1c levels (−1.9 ± 1.70% at week 24; p < 0.001) with good tolerability [20].

The VERIFY (Vildagliptin Efficacy in combination with metfoRmIn For earlY treatment of type 2 diabetes) trial was a large multicenter trial conducted across 254 clinical centers in 34 countries involving 1,598 patients with a study period of five years. The VERIFY study demonstrated that early combination therapy with vildagliptin and metformin significantly reduced the relative risk of initial treatment failure (HbA1c ≥ 7.0%) by 49% (HR 0.51, p < 0.0001) compared to the initial metformin monotherapy group. After five years, the number of patients maintaining reasonable glycemic control for an extended period was more than double in the early vildagliptin and metformin combination therapy group compared to those receiving initial metformin monotherapy with vildagliptin added at the time of metformin failure.

Furthermore, the median time to loss of glycemic control was almost doubled in the early combination group (61.9 months) versus the metformin monotherapy group (36.1 months), delaying the need for treatment intensification by more than two years [21]. In addition to delaying the time to primary treatment failure, early combination therapy reduced the risk of secondary treatment failure by 26% (HR = 0.74, p < 0.0001). This suggests a "legacy effect," where early normalization of blood glucose helps attenuate diabetes progression [10,21].

Subgroup analysis of the VERIFY trial indicated that early combination therapy with vildagliptin and metformin enhanced the achievement of glycemic targets with sustained durability and delayed the need for treatment escalation compared to initial metformin monotherapy in both patients with young-onset and late-onset diabetes [22].

A meta-analysis of 11 randomized clinical trials, including a total of 8,533 patients with T2DM, confirmed that the combination of vildagliptin and metformin results in significant reductions in FPG, HbA1c, and body weight compared to metformin alone [23].

In a study evaluating vildagliptin as an add-on therapy to metformin, the combination demonstrated a significant reduction in FPG, PPG, and HbA1c levels, with no significant effect on body weight, blood lipid parameters, or hepatorenal function [11]. Evidence also suggests that initial combination therapy provides superior glycemic outcomes compared to metformin monotherapy across a wide range of baseline HbA1c levels [24]. A clinical trial involving 1,179 treatment-naive T2DM patients with HbA1c levels of 7.5-11% showed superior glycemic control with early vildagliptin and metformin combination therapy, with a favorable gastrointestinal (GI) tolerability profile compared to metformin monotherapy [25].

In an observational study from India, where glycemic control was evaluated in 280 T2DM patients, vildagliptin and metformin combination therapy showed a robust response with a significant HbA1c reduction of 1.9% [26]. Real-world evidence from India also suggests that combining metformin with vildagliptin significantly reduces HbA1c levels compared to metformin alone. In this study, patients requiring additional oral antidiabetic drugs at follow-up were significantly fewer in the metformin plus vildagliptin arm than in the metformin-only arm [27]. Earlier evidence has suggested that the metformin IR-vildagliptin combination is associated with higher adverse events, such as gastric discomfort, acidity, nausea, and indigestion, than the metformin SR-vildagliptin combination [14]. A phase 4 study conducted in India found that the once-daily dose of vildagliptin 100 mg SR is as effective and safe as the twice-daily dose of 50 mg in reducing HbA1c, FPG, and PPG when taken in conjunction with metformin 1000 mg. The study suggests that the once-daily vildagliptin SR 100 mg formulation is bioequivalent to the twice-daily vildagliptin IR 50 mg formulation, which may result in a clinically meaningful glucose-lowering effect, reduced pill burden, and improved compliance for patients with T2DM [28]. Vildagliptin SR/metformin SR can be preferred over other second-line options, as it offers a favorable safety profile, particularly in terms of hypoglycemia risk, and may be better tolerated in patients with compromised renal function [6,7].

In our study, the FDC of vildagliptin 100 mg SR and metformin 500/1000 mg SR was effective as early combination therapy in both newly diagnosed T2DM patients and as an add-on therapy in T2DM patients uncontrolled on existing antidiabetic therapy, with a significant reduction in glycemic parameters, including HbA1c, FPG, and PPG. Subgroup analysis by age and BMI did not show any significant within-group differences. Based on the duration of T2DM, the HbA1c reduction was higher in patients with a duration of <1 year (−1.18%) compared to patients with a duration of ≥1 year (−0.71% to −0.91%). This finding reemphasizes the outcomes of the VERIFY trial, which found that initiating combination therapy early in the course of T2DM leads to better glycemic control. Further, based on the presence or absence of comorbidities, the HbA1c reduction was −1.31% in T2DM patients with comorbidities and −0.73% in those without known comorbidities. This difference is probably due to differences in baseline HbA1c levels (Table 3). Based on the subgroup analysis, it can be derived that the combination of vildagliptin 100 mg SR and metformin 500/1000 mg SR is effective across patient age, BMI, T2DM duration, and presence or absence of comorbid conditions. The study findings also indicate that the treatment was safe and well tolerated among this set of T2DM patients.

The evidence indicates that using a fixed-dose combination of vildagliptin SR and metformin SR can further help enhance treatment compliance and adherence due to once-daily dosing and a good glycemic control and tolerability profile.

Limitations

Despite the valuable insights provided by this study, several limitations must be acknowledged. The retrospective design limits causal inferences, introduces selection bias, and lacks randomization, which may impact the validity of the findings. Additionally, confounding factors such as diet, lifestyle, and medication adherence were not controlled, potentially influencing outcomes. A key limitation is the absence of a comparator group, making it difficult to assess how the FDC therapy compares to other antidiabetic treatments. Furthermore, the follow-up period of 90 days was relatively short, restricting conclusions regarding long-term efficacy and safety. While data collection from multiple centers enhances generalizability, it may also introduce variability in treatment practices. Another important limitation is the reliance on physician-documented adverse event reporting, which may have led to underreporting of minor or transient side effects. Additionally, the study did not assess cardiovascular or renal outcomes, which are critical considerations in the long-term management of T2DM.

Future research should focus on prospective, randomized controlled trials with longer follow-up durations to better evaluate the comparative effectiveness, long-term safety, and real-world adherence of FDC therapy. Future research should also include parameters for cardiovascular and renal outcomes. Additionally, studies incorporating detailed adverse event monitoring, as well as assessments of lifestyle and adherence factors, would provide a more comprehensive understanding of treatment outcomes.

## Conclusions

In conclusion, the fixed-dose combination of vildagliptin SR and metformin SR is an effective, safe, and well-tolerated treatment option for managing T2DM in Indian patients, whether newly diagnosed or inadequately controlled on other antidiabetic agents. The significant improvements in glycemic parameters and the favorable safety profile observed in this real-world study support the use of this FDC in routine clinical practice. Further studies with longer follow-up periods and larger sample sizes are warranted to confirm these findings and to explore the long-term benefits of this treatment approach.
